# Metal-free ferroelectric halide perovskite exhibits visible photoluminescence correlated with local ferroelectricity

**DOI:** 10.1126/sciadv.abo1621

**Published:** 2022-06-22

**Authors:** Taketo Handa, Ruito Hashimoto, Go Yumoto, Tomoya Nakamura, Atsushi Wakamiya, Yoshihiko Kanemitsu

**Affiliations:** Institute for Chemical Research, Kyoto University, Uji, Kyoto 611-0011, Japan.

## Abstract

Perovskite materials with tunable electronic and structural characteristics can realize various physical properties including electrical/ionic conduction, ferroelectricity, and luminescence. Integrating and coupling these properties in a single perovskite material offer new possibilities for fundamental research and applications. In particular, coupling ferroelectricity and luminescence would enable novel applications. Here, we report that the metal-free ferroelectric perovskite MDABCO (*N*-methyl-*N*′-diazabicyclo[2.2.2]octonium)–ammonium triiodide exhibits coupled superior ferroelectricity and visible photoluminescence (PL). Besides strong second-harmonic generation (SHG) associated with its ferroelectricity, MDABCO–ammonium triiodide shows long-lifetime PL at room temperature. Remarkably, the PL intensity depends strongly on the polarization of the excitation light. We found that this anisotropy is coupled to the local crystal orientation that was determined by polarization-resolved SHG. Our results suggest that the anisotropic PL property can be tuned in response to its ferroelectric state via an external field and, thereby, presents a previosuly unobserved functionality in perovskites.

## Teaser

Distinct coupling between luminescence and ferroelectric properties was found in metal-free organic perovskite material.

## INTRODUCTION

Perovskites with the crystal structure ABX_3_ (A and B are cations and X is anion) offer versatile functionalities and are promising materials for applications to electronics, photonics, and optoelectronics (*1*). What makes perovskites so attractive is their ability to realize coexistence and couplings of two (or more) important functionalities in a single material, e.g., multiferroics that combine ferroelectricity and ferromagnetism (*2*–*4*). One of the most important functionalities of the perovskite family is ferroelectricity, which has been obtained in oxide perovskites and has several important applications in actuators, sensors, and memories (*5*). More recently, the emergence of halide perovskites has enabled functionalization of another important feature (*6*); they show highly efficient luminescence (*7*–*9*), unlike oxide perovskites (*10*, *11*). Here, the ability to couple ferroelectric and luminescence properties would have various novel applications, such as electric field control of luminescence property and contactless optical reading of the ferroelectric state. However, these properties have not been realized in one perovskite material with sufficient performance. Although doping ferroelectric oxide perovskite with rare earth metals has been used to induce these features (*12*), the use of rare earth metals may limit its applications, and the host-guest configuration is disadvantageous in terms of charge carrier transport.

In this context, a recent study reported a successful synthesis of a new halide perovskite featuring superior ferroelectricity: MDABCO (*N*-methyl-*N*′-diazabicyclo[2.2.2]octonium)–NH_4_I_3_ (*13*). This material exhibits a large spontaneous polarization of 22 μC/cm^2^ and a high Curie temperature of 448 K, surpassing those of conventional oxide perovskites, such as BaTiO_3_. Furthermore, the B site is occupied by the organic NH_4_, so it is a metal-free ferroelectric. This feature may avoid the frequently discussed toxicity issues of lead-containing perovskites (*14*, *15*). Accordingly, the mechanism of its ferroelectricity has been actively studied (*16*, *17*). In addition, recent work has demonstrated excellent charge carrier transport in a similar metal-free perovskite (*18*), making them promising for optoelectronic applications. However, very little is known about its optical properties. Of high interest is the luminescence property, particularly its possible correlation with the ferroelectricity. Although it was shown in (*13*) that MDABCO-NH_4_I_3_ can emit photoluminescence (PL), the relation between the ferroelectric and PL properties has not been discussed so far. It would be interesting if a direct correlation exists between its ferroelectricity and luminescence, rather than these properties being independent.

Here, we report that MDABCO-NH_4_I_3_ is a ferroelectric luminescent material exhibiting visible PL that is directly coupled to the local crystal orientation. We prepared high-quality single crystals with crystallographically well-defined facets and performed macroscopic optical spectroscopy, microscopic polarization-resolved measurements, and theoretical analysis. MDABCO-NH_4_I_3_ shows efficient second-harmonic generation (SHG) that is associated with its ferroelectricity and a clear orange PL with a long lifetime of ~1 μs. We found that the PL intensity depends strongly on the polarization of the excitation light. Furthermore, we revealed that the PL anisotropy is correlated with a specific crystal orientation that we determined from polarization-resolved SHG. To our knowledge, this is the first observation of an anisotropic PL property in a material exhibiting an intrinsic coupling between ferroelectricity and luminescence. We clarify the microscopic origin of the anisotropic optical transition and its relation to the ferroelectricity. Our findings open new possibilities for optical probing of electronic states in ferroelectrics.

## RESULTS

### Crystal structure of MDABCO-NH_4_I_3_

**Fig. 1.**
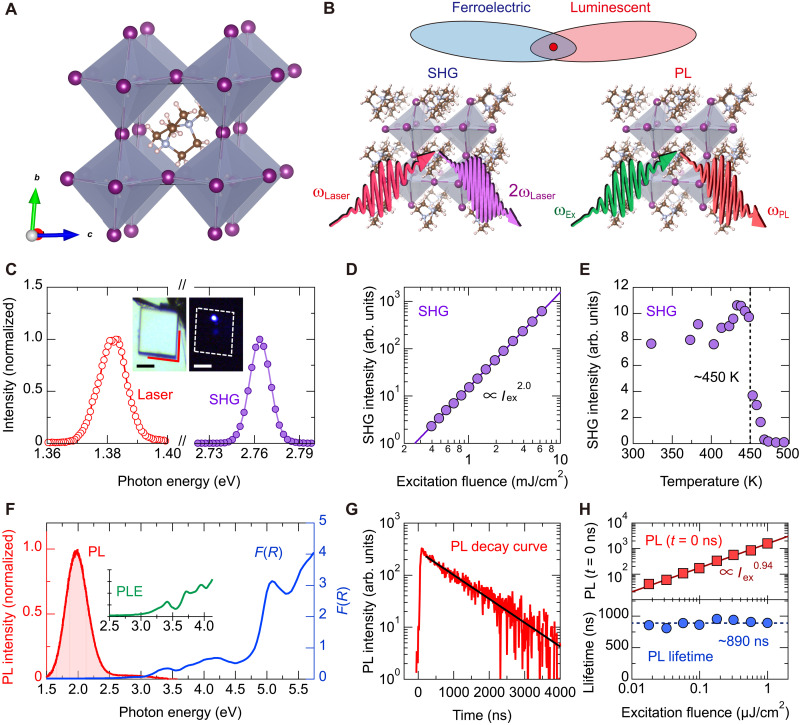
Strong SHG and visible, long-lifetime PL from metal-free ferroelectric perovskite MDABCO-NH_4_I_3_. (**A**) Crystal structure of MDABCO-NH_4_I_3_ determined by SCXRD. The NH_4_ molecules (B-site cation) are located at the vertices of the unit cell that is shown as the black solid line. (**B**) Combination of ferroelectric and luminescence properties. SHG measurements are a useful method for studying ferroelectricity. (**C**) SHG spectrum of sample under near-infrared laser excitation. The insets show optical images of the sample under white light (left) and under laser excitation with showing strong SHG (right), where scattering of the excitation laser is cut using a short-pass filter. The red lines indicate the crystal edge, at which the angle is 85°. Scale bars, 50 μm. (**D**) Excitation fluence dependence and (**E**) temperature dependence of the SHG intensity. arb. units, arbitrary units. (**F**) PL spectrum and Kubelka-Munk function, *F*(*R*). The inset shows the PL excitation spectrum monitored at 2.0 eV. (**G**) PL decay curve monitored at a photon energy of 2.0 eV, under 300-nm (4.1-eV) laser excitation. (**H**) Excitation fluence dependences of PL intensity just after photoexcitation (top) and PL lifetime (bottom).

Single crystals of MDABCO-NH_4_I_3_ were prepared on a quartz substrate by slowly evaporating a precursor solution (see Materials and Methods for details). High-quality crystals with flat surfaces were obtained at processing temperatures lower than 100°C. The crystal structure was determined by single-crystal x-ray diffraction (SCXRD) at 293 K [Fig. 1A; drawn using VESTA (*19*)]. MDABCO-NH_4_I_3_ has a rhombohedral lattice that is slightly deformed from the cubic perovskite structure along its diagonal direction. The (NH_4_)I_6_ octahedra are three-dimensionally connected via corner sharing, while large MDABCO molecular cations fill the voids. The SCXRD analysis also revealed that MDABCO-NH_4_I_3_ belongs to the trigonal space group *R*3 with lattice constants of *a*_R_ = *b*_R_ = *c*_R_ = 7.2599 Å and α_R_ = β_R_ = γ_R_ = 84.757° at room temperature. The crystal structure agrees with the previous report (*13*). Note that the subscript R of the lattice parameters indicates that the rhombohedral axis representation is used for describing the unit cell; this explicit definition is particularly important for discussing the present trigonal space group, for which rhombohedral or hexagonal lattice descriptions can be considered (see Materials and Methods) (*20*).

The MDABCO-NH_4_I_3_ sample crystallized in a rhombic form in real space (as shown below), which should reflect the rhombohedral unit cell (Fig. 1A). The angle at the crystal edge was found to be 85°, and it coincides with the lattice angle determined by SCXRD, suggesting that the rhombic crystal surface corresponds to the rhombohedral (100)_R_ or ($\bar{1}00$)_R_ facet. This assignment was further corroborated using SCXRD to determine the face index of the individual crystal (see fig. S1). An accurate determination of the crystal facet is important for a quantitative analysis of the SHG results.

As reported below, we performed several spectroscopic measurements to understand the fundamental PL and nonlinear optical properties of this ferroelectric material and then conducted detailed polarization-resolved measurements to probe the local ferroelectricity and anisotropy in the optical transition.

### Fundamental linear and nonlinear optical properties

The space group *R*3 determined above belongs to point group 3, which is one of the polar crystal classes. This result shows that MDABCO-NH_4_I_3_ is a ferroelectric with a permanent dipole at room temperature. Ferroelectrics have broken inversion symmetry and accordingly have nonzero second-order nonlinear susceptibility (*21*), giving rise to optical SHG (see Fig. 1B). The second-order nonlinear susceptibility is fundamentally related to the crystal symmetry, and its detailed study is useful for investigating, e.g., ferroelectric-paraelectric phase transition and spontaneous polarization direction in ferroelectrics (*21*, *22*). SHG measurements are, thus, an effective means for investigating ferroelectric properties in MDABCO-NH_4_I_3_.

We observed strong SHG from MDABCO-NH_4_I_3_. Figure 1C shows the SHG signal under irradiation by a near-infrared laser. The inset of Fig. 1C presents optical images under white light (left) and near-infrared excitation (right), where SHG (blue in color) can be seen even at a weak incident laser power of 600 μW and a relatively short exposure time of 100 ms. We also confirmed that the SHG intensity increases quadratically against the excitation laser fluence (Fig. 1D).

Figure 1E illustrates the temperature dependence of the SHG intensity. The SHG intensity sharply dropped at around 450 K. This temperature is in agreement with the reported ferroelectric-piezoelectric phase transition temperature of MDABCO-NH_4_I_3_ (*13*). Therefore, the SHG observed at room temperature is associated with the broken inversion symmetry that is directly related to the ferroelectricity of this material. In the ferroelectric phase, the spontaneous polarization direction is along [111]_R_ (*13*). Thus, as elaborated later, a detailed analysis of the SHG enables us to study the crystal orientation and spontaneous polarization direction.

In addition to the strong nonlinear optical response inherent to its ferroelectricity, MDABCO-NH_4_I_3_ shows clear visible PL. Figure 1F presents the optical absorption and PL spectra at room temperature. Under 300-nm (4.1-eV) laser excitation, broad PL is apparent at 2 eV.

The PL properties of MDABCO-NH_4_I_3_ were further characterized as follows. Figure 1G shows the PL decay curve. We found that this material has a long PL lifetime of around 900 ns, almost independent of the excitation fluence (Fig. 1H, bottom). The PL intensity just after photoexcitation showed a nearly linear dependence against excitation fluence (Fig. 1H, top), suggesting that exciton-like monomolecular recombination is responsible for the observed luminescence. In addition, the PL at 2 eV is considerably redshifted compared to the onset energy of the absorption and PL excitation spectra at around 3 eV (see Fig. 1F): PL thus has a large Stokes shift. Note that these PL responses are typical characteristics of recombination of self-trapped excitons, as the Supplementary Materials explain in view of the electronic structure (*23*, *24*), ionicity and lattice constants (*25*–*27*), and deformation potential (*16*, *28*, *29*). In particular, the observed long PL lifetime of ~1 μs and the characteristic absorption spectrum are similar to those of alkali iodides, typical materials where self-trapped excitons dominate the optical responses (*27*). The similarities between MDABCO-NH_4_I_3_ and alkali iodides indicate a certain contribution from the iodine atoms within the lattice (see the Supplementary Materials). Consequently, MDABCO-NH_4_I_3_ shows long-lived, visible luminescence.

### Observation of anisotropic SHG and PL

We confirmed that ferroelectricity and visible PL emission coexist in MDABCO-NH_4_I_3_ at room temperature. Moreover, if the luminescence is directly correlated with the local ferroelectricity (i.e., optical anisotropy is correlated with the direction of spontaneous polarization), then MDABCO-NH_4_I_3_ would be an exceptionally unique material. In ferroelectrics, the local crystal orientation and spontaneous polarization direction can be revealed through polarization-resolved SHG, where the angular dependence of the second-harmonic intensity is studied with respect to the excitation laser polarization and/or crystal axes (*22*, *30*, *31*). Here, we complemented the SHG technique with polarization-resolved PL measurements to directly elucidate the relation between the crystal orientation and luminescence property.

**Fig. 2.**
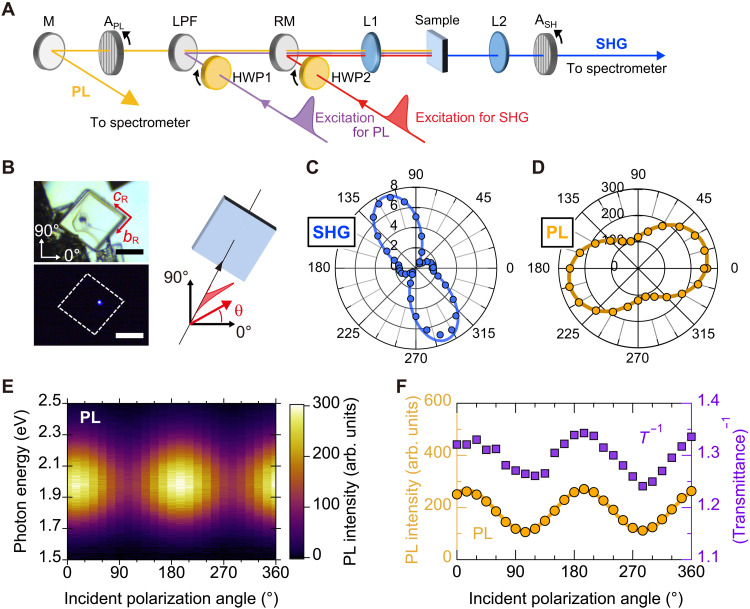
Observation of anisotropic SHG and PL from MDABCO-NH_4_I_3_ crystal. (**A**) Schematic illustration of polarization-resolved SHG and PL setup. M, mirror; RM, resettable mirror; L, lens; A_PL_ (A_SH_), polarization analyzer for PL (SHG); HWP, half-wave plate; LPF, long-pass filter. Femtosecond laser lights with two wavelengths of 300 and 897 nm served as the excitation for PL and SHG, respectively. (**B**) Optical image of typical sample used for the polarization-resolved measurements (top left) and that showing SHG under laser excitation (bottom left), together with the definition of the incident polarization angle (right). The crystallographic axes were determined by the analysis shown in Fig. 3. Scale bars, 100 μm. (**C**) Polar plots of the SHG intensity and (**D**) PL intensity versus incident polarization angle measured without placing an analyzer. Clear anisotropies are observed in the SHG and PL plots, and they are correlated with each other (see also Fig. 4). The solid curve in (C) shows the fitting result described in the main text, while that in (D) shows the fitting result using a cosine function. (**E**) Incident polarization dependence of PL spectrum. (**F**) Comparison of anisotropy in PL intensity and inverse transmittance at the excitation wavelength.

Figure 2A illustrates the setup for the polarization-resolved SHG and PL experiment. Briefly, laser lights with wavelengths of 897 and 300 nm were used as excitations for SHG and PL, respectively. Each light passed through a specific half-wave plate to control its polarization. In addition, analyzers were placed in the detection paths and were either rotated or fixed at a specific angle to resolve the polarization of the emitted light fields. The excitation beam was normally focused to the rhombohedral-like crystal facet (Fig. 2B), with a spot size of ~5 μm to resolve the local response (see Materials and Methods).

**Fig. 3.**
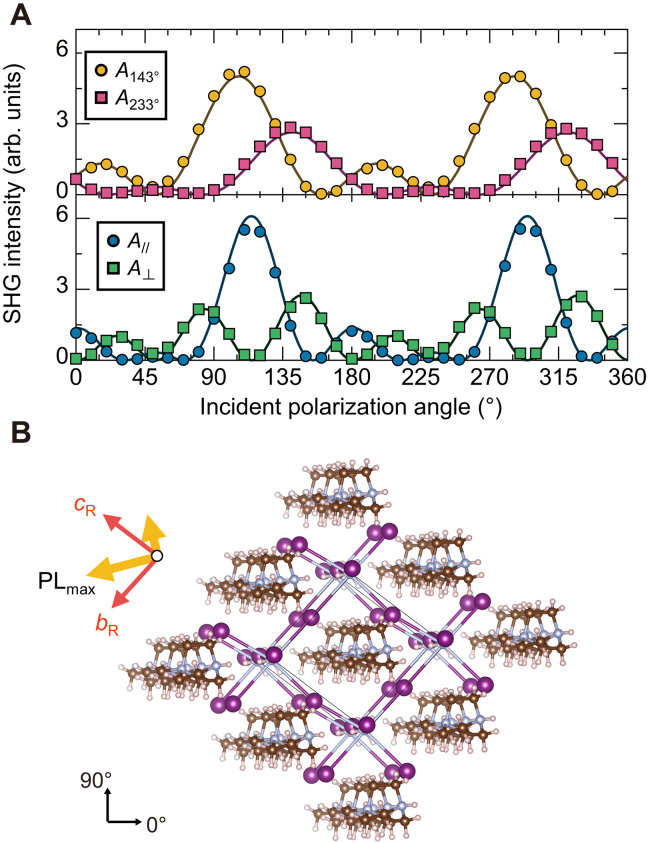
Determination of crystal orientation and its relation to PL anisotropy. (**A**) Incident polarization dependence of SHG intensity for various analyzer configurations (filled symbols) together with fitting results (solid curves). The top shows the polarization dependences measured at fixed analyzer angles, while the bottom shows those obtained by keeping the analyzer’s transmission axis parallel or perpendicular to the excitation polarization. *A* represents the transmission axis of the analyzer. (**B**) Crystallographic orientation determined by polarization-resolved SHG for the sample shown in Fig. 2B. The rhombohedral lattice vectors and the PL anisotropy directions are indicated by red and orange arrows, respectively.

Figure 2C shows a polar plot of the SHG intensity that was obtained by rotating the excitation laser polarization without placing the analyzer. Note that the polar plots reported in this study are presented so that they can be directly compared with the real-space images; e.g., see Fig. 2B. MDABCO-NH_4_I_3_ shows a clear, twofold anisotropy in SHG (Fig. 2C). The lack of higher symmetries, e.g., threefold symmetry, is consistent with the fact that the (100)_R_ or ($\bar{1}00$)_R_ facet with low symmetry was measured. This anisotropic SHG will be used for determining the local crystal orientation (Fig. 3).

Moreover, we performed polarization-resolved PL at the same position where the SHG was measured. Figure 2D shows a polar plot of the PL intensity obtained by rotating the excitation laser polarization without placing the analyzer. We observed a distinct anisotropy in the PL intensity. The PL intensity shows a twofold symmetry that is well described by a cosine function, and the maximum/minimum intensity ratio is 2.4. Neither the PL spectral shape nor the peak position depends on the excitation polarization (Fig. 2E). The angle at which the PL intensity shows the maximal peak coincides with the angle at which the SHG shows the smaller peak (Fig. 2C).

To gain a deeper understanding of the unique anisotropic PL, the polarization dependence of the transmittance at the excitation wavelength of 300 nm was measured. The result revealed that the inverse transmittance well correlates with the excitation polarization–dependent PL (Fig. 2F). This result indicates that the observed anisotropic PL is dominated by the anisotropy in the linear absorption process at the excitation wavelength. We further measured the emission polarization dependence of the PL under fixed excitation polarization by placing the analyzer in the detection path and by rotating it (fig. S3). The PL intensity exhibited no dependence on the analyzer angle, showing that the PL emission is unpolarized. In addition, we confirmed that the transmittance at the PL peak energy does not depend on the light polarization (fig. S4). Consequently, we conclude that MDABCO-NH_4_I_3_ exhibits a polarization-sensitive linear optical absorption at the excitation wavelength and an unpolarized visible PL emission after photocarrier relaxation. This is the first observation of anisotropic PL property in an intrinsic ferroelectric material. This phenomenon has not been reported until now because of the lack of luminescent ferroelectrics.

### Optical anisotropy and crystal orientation

Now, let us discuss the relation between the observed PL anisotropy and the local crystal orientation by examining the results of polarization-resolved SHG experiments with the analyzer being placed on the detection path (*30*, *31*). The excitation polarization dependences of SHG were measured by keeping the analyzer’s transmission axis parallel or perpendicular to the excitation polarization (Fig. 3A, bottom) and by fixing the analyzer at certain angles (Fig. 3A, top), which were subsequently analyzed by deriving theoretical expressions as the following.

For MDABCO-NH_4_I_3_, which has a *C*_3_ rotational symmetry along the [111]_R_ direction (Fig. 1A), the symmetry analysis (*21*) reveals that the nonvanishing tensor elements of the second-order nonlinear susceptibility *d_ij_* inducing SHG are *d*_11_ = −*d*_12_ = −*d*_26_, *d*_15_ = *d*_24_ = *d*_31_ = *d*_32_, *d*_22_ = −*d*_21_ = −*d*_16_, and *d*_33_. The product of *d_ij_* and the electric field *E_l_* (*l* = *x*, *y*, *z*) then determines the nonlinear polarization and, subsequently, SHG (*21*). These expressions are based on Cartesian coordinates, while the optical beam is incident on a rhombohedral crystal facet whose relation to Cartesian coordinates is not straightforward (see Materials and Methods and the Supplementary Materials) (*32*, *33*). To overcome the difficulty in expressing the in-plane electric field within the rhombohedral facet using Cartesian coordinates, we introduced coordinate transformation matrices (*20*) and derived a useful formula for transforming the electric field expressions between different coordinates (see Materials and Methods). The obtained electric field components of *E_l_* and the nonlinear susceptibility *d_ij_* yield the nonlinear polarization **P**_**2ω**_, which consequently gives the SHG intensity via *I*_SH_ ∝ (**P**_**2ω**_ **· A**)^2^, where **A** represents the transmission axis of the analyzer. The theoretical expressions were fitted to the experimental results using a global fitting procedure.

In Fig. 3A, the solid curves show the best-fit results based on the expressions obtained for the ($\bar{1}00$)_R_ facet. The theoretical expressions match quite well with the experimental results. The analysis gives a constraint equation that determines the relative relationship between the *d_ij_* values (see the Supplementary Materials). Moreover, it enables us to determine the local crystal orientation, as depicted in Fig. 3B.

In Fig. 3B, in addition to the lattice vectors, the direction of the PL anisotropy is indicated with orange arrows. The peak of the PL excitation is at an angle of 32.5° from the *b*_R_ axis (this is close to the direction along the spontaneous polarization [111]_R_; 42.4°). A closer look at the figure reveals that the direction of maximum PL intensity is parallel to one of the NH_4_─I─NH_4_ bonds. This is consistent with our earlier discussion that the excitation light can be considered to excite electronic states formed by iodine atoms (see also the Supplementary Materials). The stronger optical transitions along the particular bonding direction can be attributed to a slight modulation of the electronic state because this bond is nearly parallel to the spontaneous polarization direction. These results show an intrinsic coupling between ferroelectricity and luminescence.

**Fig. 4.**
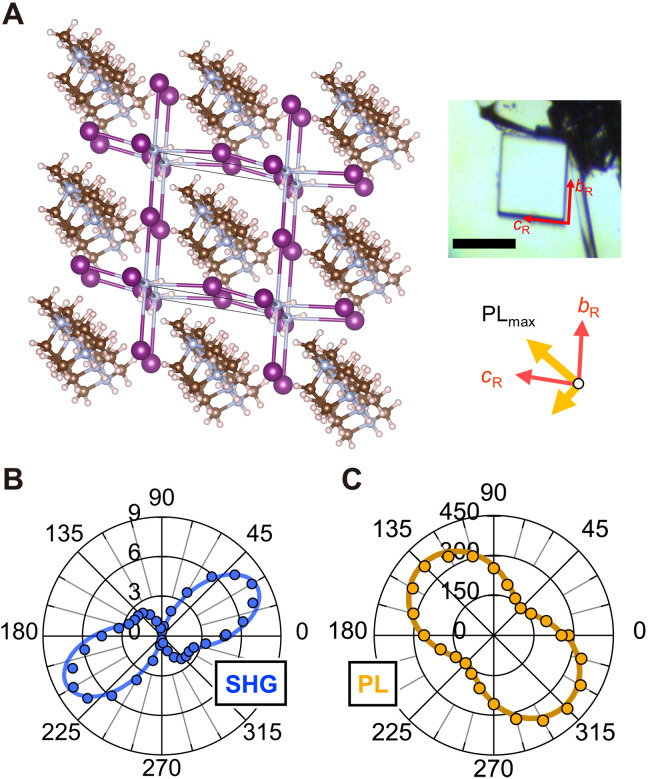
Polarization-resolved SHG and PL for a different crystal. (**A**) Crystallographic orientation determined by polarization-resolved SHG. The inset is an optical image of the sample. Scale bar, 100 μm. (**B**) Polar plots of SHG intensity and (**C**) PL intensity versus incident polarization angle, measured without the analyzer. The solid curves in (B) and (C) are the fitting results as explained in Fig. 2. This crystal shows almost the same features as those in Figs. 2 and 3, except for that the SHG anisotropy exhibits an opposite rotational direction (see Fig. 2C). This demonstrates that polarization-resolved SHG can distinguish the difference between the (100)_R_ and ($\bar{1}00$)_R_ planes.

Last, we performed the same measurements on different samples. As shown in Fig. 4 (A and B), another crystal exhibited very similar anisotropy in SHG, while one can see that an inversion of the rotational direction occurs; in Fig. 2C, a weak SHG peak appears at 78° from the strong SHG peak in the counterclockwise direction, while in Fig. 4B, it appears in the clockwise direction. This result shows that SHG certainly distinguishes the (100)_R_ and ($\bar{1}00$)_R_ surfaces (*22*). In the crystal shown in Fig. 4A, the [100]_R_ axis is oriented out of the paper plane. One can also see that the PL excitation shows a similar, distinct anisotropy whose relation to the SHG anisotropy is identical to that of the previous sample (Fig. 4C). The consistent results in Figs. 2 and 4 not only confirm the observation being the intrinsic effect but also verify the above conclusion on the relation of the anisotropic optical transition to the spontaneous polarization direction.

## DISCUSSION

Together, our results demonstrate that MDABCO-NH_4_I_3_ is a luminescent ferroelectric material exhibiting optical absorption sensitive to incoming light polarization, which is attributed to the specific bonding direction. Thus, the PL intensity can be uniquely tuned depending on the relative relation between the excitation light polarization and lattice configuration. It has been reported that the ferroelectricity of MDABCO-NH_4_I_3_ mainly originates from a displacive-type mechanism (*29*). Therefore, modulation of the spontaneous polarization through application of an external electric field will displace lattice ions. Accordingly, an external field could be used to control the anisotropy in optical absorption and, thereby, manipulate the PL property. Furthermore, because of the ferroelectricity of this material, modulations of the optical properties would be persistent even after the external field is removed. Our findings on this unique combination of superior ferroelectricity and visible luminescence in the new type of perovskite will expand the horizons of materials science and optoelectronics.

## MATERIALS AND METHODS

### Sample preparation

To prepare the MDABCO-NH_4_I_3_, 1 ml of 50% H_3_PO_2_ aqueous solution and 1.3 ml of 57% HI aqueous solution were added to 1.5 ml of water. A total of 635 mg of MDABCO-I (2.5 mmol) and 363 mg of NH_4_I (2.5 mmol) were dissolved in this solution at 100°C. The reaction solution was dropped on a quartz substrate, which was then covered with a petri dish, and MDABCO-NH_4_I_3_ crystals were annealed with a hotplate (100°C for 20 min). The petri dish was removed, and the sample was annealed with a hotplate (100°C for 10 min). Subsequently, the sample was transferred to an argon-filled glove box, where the sample was mounted in a cryostat for the optical measurements.

### Single-crystal x-ray diffraction

Diffraction data were recorded at 293 K on a Bruker single-crystal charge-coupled device x-ray diffractometer (SMART APEX II ULTRA) with Mo Kα radiation (λ = 0.71073 Å) and a graphite monochromator. A total of 3430 reflections were measured with a maximum 2θ angle of 51.0°, of which 1326 were independent reflections. The structure was solved using direct methods [SHELXS-97 (*34*)] and was refined by full-matrix least squares on *F*^2^ [SHELXL-97 (*34*)]. All non-hydrogen atoms were refined anisotropically. All hydrogen atoms were placed using AFIX instructions. The crystal data are as follows: C_7_H_20_I_3_N_3_; formula weight = 526.96, crystal size of 0.13 mm by 0.20 mm by 0.30 mm, trigonal, *R*3, *a* = 7.2599(8) Å, α = 84.7570(10)°, *V* = 378.11(13) Å^3^, *Z* = 1, and *D*_c_ = 2.314 g cm^−3^. The refinement converged to *R*_1_ = 0.0402, w*R*_2_ = 0.1132 [*I* > 2 s (I)], and the goodness of fit was = 1.353. Crystallographic data have been provided in the Cambridge Crystallographic Data Centre (CCDC) [CCDC-2157903].

### SHG and PL measurements

The SHG measurements were performed using a femtosecond laser light with a fundamental wavelength of 897 nm, which was obtained from an ytterbium-doped potassium gadolinium tungstate (Yb:KGW) regenerative amplifier with a repetition rate of 200 kHz and an optical parametric amplifier (OPA). The laser light was passed through a variable neutral density filter, a linear polarizer, and a half-wave plate, reflected by a resettable metallic mirror, and focused by a lens onto the sample in a vacuum (Fig. 2A). The emitted SHG signal was recorded using a fiber spectrometer. A short-pass filter was placed on the detection path to block the excitation laser, while a wire grid linear polarizer (analyzer) was placed to resolve the angular dependence of the emitted SHG intensity. When performing the polarization-resolved SHG, we acquired an SHG image to confirm that no observable domain structure existed within or near the measured region (see fig. S5). The homogeneous SHG intensity within the area of about 25 μm by 25 μm suggests that the spontaneous polarization direction was uniform within this area. This is consistent with the previous study using piezoelectric force microscopy, in which a uniform polarization direction was shown with an area measuring several tens of micrometers (*13*). In addition, we confirmed that the quartz substrate showed no SHG signal within the laser fluence.

As the excitation for the PL measurements, we used a femtosecond laser light with a wavelength of 300 nm, which was obtained by frequency doubling the output from the OPA using a beta barium borate crystal. The laser beam was passed through a variable neutral density filter, a linear polarizer, and a half-wave plate for ultraviolet light, reflected by a long-pass filter, and focused onto the sample by a lens. The PL from the sample was collected and collimated using the same lens as for the excitation. The collimated PL was subsequently passed through the long-pass filter and a wire grid linear polarizer (analyzer). The PL spectrum was recorded using a nitrogen-cooled charge-coupled device camera equipped with a monochromator. The spectral sensitivity of the setup and its polarization dependence due to the rotation of the analyzer were corrected by using a standard white light source and acquiring its spectrum at different analyzer angles.

The radii of the 897- and 300-nm laser lights were 4.9 and 5.3 μm at the sample position, respectively. Because the excitation wavelengths for PL and SHG were rather different, two different lenses (with the same focal length) were used to achieve the best focus at the sample position for each excitation condition. The optical images of the sample were obtained with a commercial complementary metal-oxide semiconductor (CMOS) camera. This CMOS camera was also used to ensure that the SHG and PL measurements were performed at the same position. The above polarization-resolved measurements were performed at room temperature and in a vacuum. During the measurements, the material showed a stable PL emission, which can be confirmed in Figs. 2D and 4C by comparing the PL intensities at 0° and 360°.

The temperature dependence of the SHG intensity was measured by placing the sample in a controllable-temperature isothermal sample analysis stage filled with nitrogen. Time-resolved PL was measured using a streak camera. The PL excitation spectrum was obtained from the sample in air using a commercial spectrofluorometer. The optical absorption spectrum was obtained by measuring the diffuse reflectance *R*(ℏω) and transforming *R*(ℏω) into a Kubelka-Munk function that is proportional to the absorbance: *F*(ℏω) = [1 − *R*(ℏω)]^2^/2*R*(ℏω).

### Coordinate system to describe trigonal MDABCO-NH_4_I_3_

The space group *R*3 of MDABCO-NH_4_I_3_ is one of the seven trigonal groups, where one may use either a rhombohedral axis description (primitive cell) or hexagonal axis description (triple hexagonal cell) (*20*). The former description constitutes the primitive cell, while the latter is convenient for seeing the threefold rotational axis. In the present work, because the sample crystallizes in a rhombic form (see Fig. 1C, inset), the rhombohedral description is much more convenient for specifying the crystal facet and orientation.

In the rhombohedral description, the crystal is represented by a lattice constant *a*_R_ (= *b*_R_ = *c*_R_) and lattice angle α_R_ (= β_R_ = γ_R_ ≠ 90°), while, in the hexagonal description, the crystal is represented by lattice constants *a*_H_ = *b*_H_ ≠ *c*_H_ and lattice angles α_H_ = β_H_ = 90° and γ_H_ = 120°. The rhombohedral description is convenient for describing the crystal and relevant physical properties in a rhombohedral coordinate system with rhombohedral lattice vectors (**A**_**R**_, **B**_**R**_, and **C**_**R**_). The hexagonal coordinate system is good for discussing physical properties in terms of hexagonal lattice vectors (**A**_**H**_, **B**_**H**_, and **C**_**H**_). Another description is the conventional Cartesian coordinates represented with unit vectors (**e**_**x**_, **e**_**y**_, and **e**_**z**_).

Note that the second-order nonlinear susceptibility is conventionally defined in Cartesian coordinates. The conventional expressions (*21*) for the relations between tensor elements are relevant only when the symmetry axes of the crystal are appropriately aligned with respect to Cartesian coordinates. Thus, when representing the electric field within the rhombohedral crystal facet (see Fig. 1C) using Cartesian coordinates (to analyze the SHG results), one should ensure that the Cartesian coordinates for representing the electric fields are in an appropriate relation with respect to the rhombohedral coordinates of the crystal.

The relative relations between the coordinate systems are illustrated in fig. S6. Specifically, the relation between the rhombohedral and hexagonal coordinate systems can be expressed using the lattice vectors: **A**_**H**_ = **A**_**R**_ − **B**_**R**_, **B**_**H**_ = **B**_**R**_ − **C**_**R**_, and **C**_**H**_ = **A**_**R**_ + **B**_**R**_ + **C**_**R**_, where the obverse setting is adopted (*20*). Subsequently, following convention (*33*), the relation between the hexagonal and Cartesian coordinate systems can be defined by taking the threefold rotational axis (**C**_**H**_) to be parallel to **e**_**z**_ (**C**_**H**_//**e**_**z**_) and **A**_**H**_ to be parallel to **e**_**x**_ (**A**_**H**_//**e**_**x**_), while the remaining **e**_**y**_ is determined in a way that right-handed coordinates are formed. A clear specification of the coordinate system used and its relation to Cartesian coordinates are essential for studying crystals with lower symmetry. Note as well that the present material is well described in rhombohedral coordinates, where the lattice vectors are not orthogonal to each other. Therefore, using Euler’s rotation procedure alone, which is commonly used for solids with orthogonal lattices such as cubic, tetragonal, and orthorhombic crystal, is not sufficient for the analysis.

### Analyzing SHG from a trigonal material described by rhombohedral axes

The SHG from a material with broken inversion symmetry is described with a nonlinear polarization that is determined by the second-order nonlinear susceptibility (third-rank tensor) and incident electric field. Following convention, the nonlinear polarization **P**_**2ω**_ is given by (*21*)

$$P_{2\omega }=(\begin{array}{c} P_{x}\\ P_{y}\\ P_{z} \end{array})=(\begin{array}{cccccc} d_{11} & d_{12} & d_{13} & d_{14} & d_{15} & d_{16}\\ d_{21} & d_{22} & d_{23} & d_{24} & d_{25} & d_{26}\\ d_{31} & d_{32} & d_{33} & d_{34} & d_{35} & d_{36} \end{array})(\begin{array}{c} E_{x}^{2}\\ E_{y}^{2}\\ E_{z}^{2}\\ 2E_{y}E_{z}\\ 2E_{z}E_{x}\\ 2E_{x}E_{y} \end{array})$$ (1)

Here, the conventional contracted notation *d_ij_* is adopted and a constant factor of two is omitted. The electric fields are denoted by *E_l_* (*l* = *x*, *y*, *z*) and are based on Cartesian coordinates. For a crystal with *C*_3_ symmetry, the nonvanishing tensor elements are as follows (*21*): *d*_11_ = −*d*_12_ = −*d*_26_, *d*_15_ = *d*_24_ = *d*_31_ = *d*_32_, *d*_22_ = −*d*_21_ = −*d*_16_, and *d*_33_. Here, it is assumed that Kleinman’s symmetry holds because the photon energies of the fundamental and emitted second harmonics are well below the strong absorption peak of this material at 5 eV, yielding *d*_14_ = 0 and *d*_31_ = *d*_15_. This assumption admits a good explanation of the experimental results (see Fig. 3A). Note that the above relations for *d_ij_* are obtained under a certain symmetry operation, where the *z* axis in Cartesian coordinates is taken as being parallel to [111]_R_ (or [001]_H_), which constitutes the threefold rotational symmetry axis (*32*). Accordingly, we have the following expression for describing the nonlinear polarization

$$P_{2\omega }=(\begin{array}{c} P_{x}\\ P_{y}\\ P_{z} \end{array})=(\begin{array}{c} d_{11}(E_{x}^{2}-E_{y}^{2})+2d_{15}E_{z}E_{x}-2d_{22}E_{x}E_{y}\\ -d_{22}(E_{x}^{2}-E_{y}^{2})+2d_{15}E_{y}E_{z}-2d_{11}E_{x}E_{y}\\ d_{15}(E_{x}^{2}+E_{y}^{2})+d_{33}E_{z}^{2} \end{array})$$ (2)

The electric field in this equation is expressed in Cartesian coordinates. Thus, the electric field incident on the rhombohedral (100)_R_ [or ($\bar{1}00$)_R_] plane must be expressed in Cartesian coordinates. This can be done by applying the following transformation operation. Note that one may apply the transformation either to the electric field vector or to the second-order nonlinear susceptibility, while the former approach is much more convenient for the present case (*33*).

Let us denote the basis vectors for the rhombohedral coordinate system by **a**_**R**_, **b**_**R**_, and **c**_**R**_. Accordingly, the in-plane electric field on the (100)_R_ facet can be written as **E** = (cos θ − sin θ/tan α) **b**_**R**_ + (sin θ/sin α) **c**_**R**_, where α represents the lattice angle of 84.75° and θ = 0° corresponds to the **b**_**R**_ direction. The basis vectors **a**_**R**_, **b**_**R**_, and **c**_**R**_ are parallel to [100]_R_, [010]_R_, and [001]_R_, respectively. The amplitude of the incident electric field is normalized for simplicity. The terms including α arise because the lattice angle is not 90° in the present crystal; when α is taken to be 90° as in conventional orthogonal systems, one can confirm that the electric field is simply represented by **E** = cos θ **b**_**R**_ + sin θ **c**_**R**_.

We will use the following form to represent **E**, which is a product of row and column vectors and is useful for coordinate transformations

$$\begin{array}{c} E=(a_{R},b_{R},c_{R})(\begin{array}{c} A_{R}\\ B_{R}\\ C_{R} \end{array})=(a_{R},b_{R},c_{R})P_{R\to x,y,z}Q_{R\to x,y,z}(\begin{array}{c} A_{R}\\ B_{R}\\ C_{R} \end{array})\\ =(e_{x},e_{y},e_{z})(\begin{array}{c} (A_{R}-B_{R})/2\\ (A_{R}+B_{R}-2C_{R})/3\\ (A_{R}+B_{R}+C_{R})/3\gamma \end{array}) \end{array}$$ (3)

On the left-hand side, the electric field **E** is expressed in rhombohedral coordinates. Here, for the sake of obtaining a general formula, the electric field components along each basis vector are represented by *A*_R_, *B*_R_, and *C*_R_; hence, it reads for the present case as *A*_R_ = 0, *B*_R_ = cos θ − sin θ/tan α, and *C*_R_ = sin θ/sin α. The expression can be further rewritten as the equation in the middle using the transformation matrix *P*_R → *x*,*y*,*z*_, which transforms the rhombohedral coordinate system into the Cartesian coordinate system, and its inverse matrix *Q*_R → *x*,*y*,*z*_. The transformation matrix *P*_R → *x*,*y*,*z*_ is given by the product of the transformation matrices *P*_R → H_ and *P*_H → *x*,*y*,*z*_, which express the transformation from the rhombohedral to hexagonal coordinates and that from the hexagonal to Cartesian coordinates, respectively (*20*)

$$\;P_{R\to H}=(\begin{array}{ccc} 1 & 0 & 1\\ -1 & 1 & 1\\ 0 & -1 & 1 \end{array})$$ (4a)

$$P_{H\to x,y,z}=(\begin{array}{ccc} 1 & 1/2 & 0\\ 0 & 1 & 0\\ 0 & 0 & \gamma \end{array})$$ (4b)

Here, γ = |**A**_**H**_|/|**C**_**H**_| = $2\text{sin}(\frac{\alpha }{2})/(3\sqrt{1-\frac{4}{3}{\text{sin}}^{2}(\frac{\alpha }{2})})$ is the ratio between the lattice constants in the hexagonal axes description. The relative geometrical relationship of each coordinate is explained above and is illustrated in fig. S6. Here, one can confirm that the determinant of the transformation matrix *P*_R → H_ is 3, which reflects that the cell volume described in hexagonal coordinates is three times that in primitive rhombohedral coordinates.

In Eq. 3, the term (**a**_**R**_, **b**_**R**_, **c**_**R**_)*P*_R → *x*,*y*,*z*_ in the middle represents the new basis vectors after the coordinate transformation, i.e., the basis vectors in Cartesian coordinates (**e**_**x**_, **e**_**y**_, **e**_**z**_). Therefore, the concomitant term, *Q*_R → *x*,*y*,*z*_ (*A*_R_, *B*_R_, *C*_R_)*^T^* (*T* denotes the transpose), represents electric field components in the transformed Cartesian coordinates: *Q*_R → *x*,*y*,*z*_ (*A*_R_, *B*_R_, *C*_R_)*^T^* = (*E_x_*, *E_y_*, *E_z_*)*^T^*. Subsequent calculations using the actual matrices in Eqs. 4a and 4b result in the expression on the right-hand side of Eq. 3. In other words, we have derived a formula that directly converts the expressions of the electric field described in the different coordinate systems; for the present case, it converts from rhombohedral to Cartesian coordinates. For the rhombohedral (100)_R_ plane, *A*_R_ = 0, *B*_R_ = cos θ − sin θ/tan α, and *C*_R_ = sin θ/sin α, which yields expressions in Cartesian coordinates: *E_x_* = − (cos θ − sin θ/tan α)/2, *E_y_* = [cos θ − sin θ (1/tan α + 2/sin α)]/3, and *E_z_* = [cos θ − sin θ (1/tan α − 1/sin α)]/3γ. Substituting these expressions into Eq. 2 yields the nonlinear polarization **P**_**2ω**_. In addition, the transmission direction of the analyzer is expressed as **A**_**//**_ = (cos θ − sin θ/tan α) **b**_**R**_ + (sin θ/sin α) **c**_**R**_ for the parallel configuration and **A**_**⊥**_ = (−sin θ − cos θ/tan α) **b**_**R**_ + (cos θ/sin α) **c**_**R**_ for the perpendicular configuration (see Fig. 3), which can be similarly converted into Cartesian coordinates. Consequently, the inner product of the nonlinear polarization and the analyzer vector gives the theoretical expression for the SHG intensity *I*_SH_ ∝ (**P**_**2ω**_ · **A**)^2^, which was used for fitting the experimental data. The advantage of the above procedure is its simplicity in performing the coordinate transformation. The rhombohedral crystal in the present work has a lattice angle not equal to 90°, wherein it is not straightforward to convert the electric field expression. Also complicated is the relation between the directions of the basis vectors of the rhombohedral crystal coordinates and the Cartesian coordinates. The above procedure provides a more intuitive way of making coordinate transformations and allows for a straightforward conversion between the different coordinates based on the explicit matrices that have been provided in the literature (*20*).

## Supplementary Materials

### This PDF file includes:

Supplementary Text Figs. S1 to S6 References
