# Supplementary material for: Metal-free ferroelectric halide perovskite exhibits visible photoluminescence correlated with local ferroelectricity
Source: Sci Adv. 2022 Jun 22;8(25):eabo1621. doi: 10.1126/sciadv.abo1621 (PMC9217093; doi:10.1126/sciadv.abo1621)
Supplement: Supplementary file 1 — Supplementary Text Figs. S1 to S6 References [file sciadv.abo1621_sm.pdf]

Science Advances
AAAS

Supplementary Materials for

Metal-free ferroelectric halide perovskite exhibits visible photoluminescence correlated with local ferroelectricity

Taketo Handa *et al.*

Corresponding author: Yoshihiko Kanemitsu, kanemitu@scl.kyoto-u.ac.jp

Sci. Adv. **8**, eabo1621 (2022)
DOI: 10.1126/sciadv.abo1621

This PDF file includes:

Supplementary Text
Figs. S1 to S6
References

Supplementary Text

Origin of the Stokes-shifted PL in MDABCO-NH_4I_3

The broad PL observed at 2 eV is considered, as follows, to be due to recombination of self-trapped excitons. The PL at 2 eV is considerably red shifted from the absorption edge above 3 eV (see Fig. 1F in the main text). This large Stokes shift together with the Gaussian line shape indicate excited-state reorganization through self-trapping of photogenerated excitons. The formation of self-trapped excitons is known to be mainly facilitated by short-range electron-acoustic phonon interactions via a deformation potential (*35*). The magnitude of the deformation potential interaction is inversely proportional to the square root of the sound speed in the material, and recent work revealed that MDABCO-NH_4I_3 has a small bulk modulus (i.e., MDABCO-NH_4I_3 is a highly deformable material) (*16*), which corresponds to a low speed of sound in this material. Therefore, the short-range interactions in MDABCO-NH_4I_3 are expected to be strong, which should give rise to self-trapping of excitons. In addition, PL measurements were performed at room temperature, where the material is in the ferroelectric phase. This should lead to strong piezoelectric-type electron-acoustic phonon interactions, which can also promote self-trapping of excitons (*28*). Moreover, first-principles calculations for MDABCO-NH_4I_3 have revealed a very flat band structure around the valence band maximum that is composed of iodine *p* orbitals (*24*), suggesting the hole trapping as the dominating trapping process. The above microscopic considerations explain that the observed PL originates from recombination of self-trapped excitons.

Second-order nonlinear susceptibility tensor

Using the formula derived in the Materials and Methods section, the following expressions can be obtained as the SHG intensity for an analyzer angle of 0 or 90 degrees from the b_R axis for different configurations (for example, parallel and perpendicular): $(d_{11} + 12.11d_{15} - 5.11d_{22} + 2.43d_{33})^2$ and $(-1.62d_{11} + 15.1d_{15} - 8.13d_{22} + 1.84d_{33})^2$ for 0 and 90 degrees under the parallel configuration; $(-5.45d_{11} - 2.05d_{15} + 2.7d_{22} + 2.21d_{33})^2$ and $(-6.37d_{11} - 0.27d_{15} - 3.13d_{22} - 2.0d_{33})^2$ for 0 and 90 degrees under the perpendicular configuration. The relative relation between the tensor elements d_{ij} should be determined so that these expressions are constant values. Indeed, the fitting of the SHG data in Fig. 3A fulfills this condition. One can also confirm, by checking the SHG intensity at specific angle in different configurations (Fig. 3A), that the obtained results satisfy the above expressions.

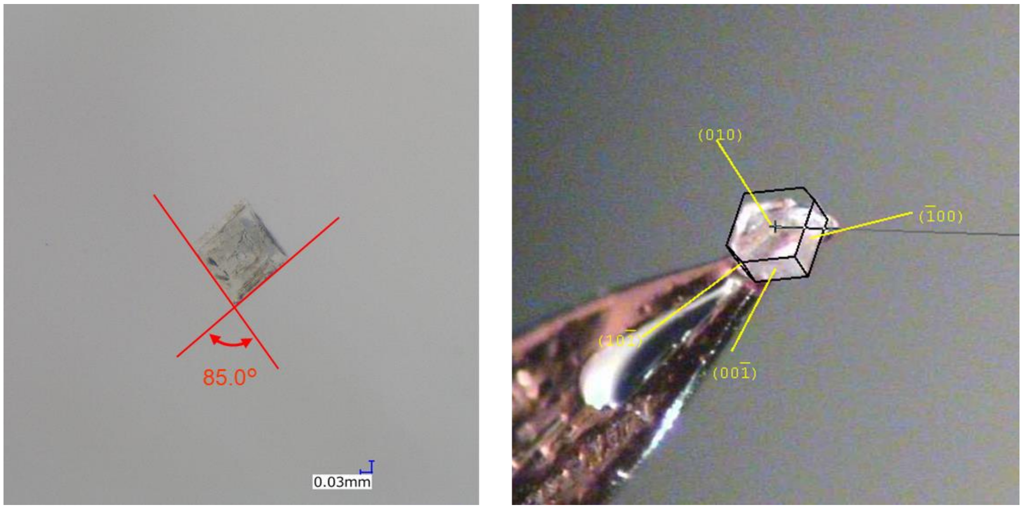

Fig. S1. Face index determination. (Left) Optical image of a representative sample and (right) determination of the face index by single-crystal XRD (SCXRD). The SCXRD result indicates that the top surface is the rhombohedral $(010)_R$ plane. In the present trigonal lattice expressed in rhombohedral axes description, the $(100)_R$, $(010)_R$, and $(001)_R$ crystal surfaces are crystallographically equivalent, $\{100\}_R$. Additionally, it is possible to distinguish between the $(100)_R$ and $(\bar{1}00)_R$ planes. Therefore, the result shows that the observed rhombic surface with an edge angle of 85° is the $(100)_R$ or $(\bar{1}00)_R$ plane.

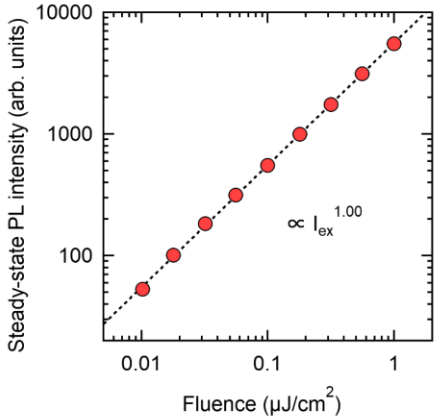

Fig. S2. Excitation fluence dependence of the steady-state PL intensity at 2 eV. The result shows the linear dependence against the excitation fluence.

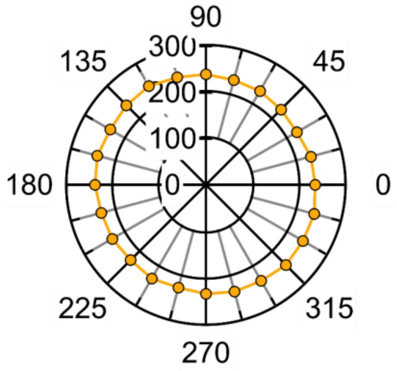

Fig. S3. Polar plot of the PL intensity at 2 eV versus the analyzer polarization angle for the sample studied in Figs. 2 and 3 in the main text. The angle shown here corresponds to the real-space definition in Fig. 2B. The polarization of the excitation laser is set to be 14.4 deg. This result shows that the PL emission is unpolarized.

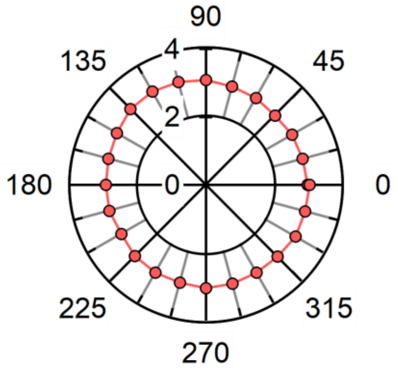

Fig. S4. Polar plot of the transmitted light intensity at 600 nm versus incident polarization angle for a typical sample. We measured the transmittance of MDABCO-NH_4I_3 at the PL emission wavelength (600 nm). The result confirms that the transmittance is independent of the light polarization, meaning that the PL anisotropy observed in Fig. 2D results from the anisotropy in the absorption process at th excitation wavelength.

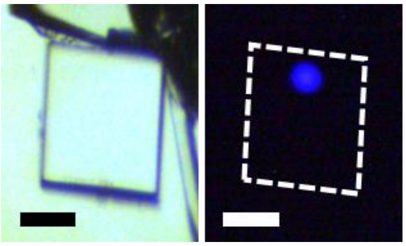

Fig. S5. SHG image that suggests the homogeneous distribution of spontaneous polarization direction. Optical images under white light (left) and under near-infrared excitation laser resulting in SHG, where the excitation laser was blocked using a short-pass filter (right). The scale bars are 50 µm. The SHG image was of the same sample as in Fig. 1C, while in Fig. S5, the spot size of the excitation laser was made larger. In this way, the spatial distribution of SHG was measured in a region larger than the actual spot size for the polarization-resolved measurement. The result indicated that the spatial distribution in the SHG intensity was homogeneous in an area of about 25 × 25 μm^2.

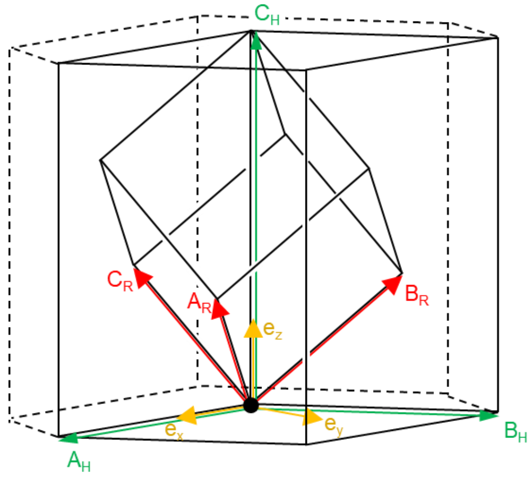

Fig. S6. Illustration of the relation between rhombohedral, hexagonal, and Cartesian coordinates in the trigonal crystal system. The rhombohedral lattice vectors (A_R, B_R, C_R) are indicated by red arrows, the hexagonal lattice vectors (A_H, B_H, C_H) by green arrows, and Cartesian unit vectors (e_x, e_y, e_z) by orange arrows. The relation between the rhombohedral and hexagonal coordinates is determined by one of the obverse settings (*20*): $A_H = A_R – B_R$, $B_H = B_R – C_R$, and $C_H = A_R + B_R + C_R$. The relation between the hexagonal and Cartesian coordinate systems is defined according to the convention (*33*): the three-fold rotational axis (C_H) is taken to be parallel to e_z (C_H//e_z) and A_H to be parallel to e_x (A_H //e_x), while the remaining e_y is determined in a way that right-handed coordinates are formed.

REFERENCES AND NOTES

1. M. Imada, A. Fujimori, Y. Tokura, Metal-insulator transitions. *Rev. Mod. Phys.* **70**, 1039–1263 (1998).

2. N. A. Spaldin, M. Fiebig, The renaissance of magnetoelectric multiferroics. *Science* **309**, 391–392 (2005).

3. W. Eerenstein, N. D. Mathur, J. F. Scott, Multiferroic and magnetoelectric materials. *Nature* **442**, 759–765 (2006).

4. Y. Tokura, Multiferroics as quantum electromagnets. *Science* **312**, 1481–1482 (2006).

5. A. S. Bhalla, R. Guo, R. Roy, The perovskite structure—A review of its role in ceramic science and technology. *Mater. Res. Innov.* **4**, 3–26 (2000).

6. S. D. Stranks, H. J. Snaith, Metal-halide perovskites for photovoltaic and light-emitting devices. *Nat. Nanotechnol.* **10**, 391–402 (2015).

7. Y. Kanemitsu, T. Handa, Photophysics of metal halide perovskites: From materials to devices. *Jpn. J. Appl. Phys.* **57**, 090101 (2018).

8. Y. Yamada, T. Nakamura, M. Endo, A. Wakamiya, Y. Kanemitsu, Photocarrier recombination dynamics in perovskite $CH_3NH_3PbI_3$ for solar cell applications. *J. Am. Chem. Soc.* **136**, 11610–11613 (2014).

9. F. Deschler, M. Price, S. Pathak, L. E. Klintberg, D. D. Jarausch, R. Higler, S. Hüttner, T. Leijtens, S. D. Stranks, H. J. Snaith, M. Atatüre, R. T. Phillips, R. H. Friend, High photoluminescence efficiency and optically pumped lasing in solution-processed mixed halide perovskite semiconductors. *J. Phys. Chem. Lett.* **5**, 1421–1426 (2014).

10. D. Kan, T. Terashima, R. Kanda, A. Masuno, K. Tanaka, S. Chu, H. Kan, A. Ishizumi, Y. Kanemitsu, Y. Shimakawa, M. Takano, Blue-light emission at room temperature from Ar^+-irradiated $SrTiO_3$. *Nat. Mater.* **4**, 816–819 (2005).

11. Y. Yamada, H. Yasuda, T. Tayagaki, Y. Kanemitsu, Temperature dependence of photoluminescence spectra of nondoped and electron-doped $SrTiO_3$: Crossover from Auger recombination to single-carrier trapping. *Phys. Rev. Lett.* **102**, 247401 (2009).

12. X. Tian, Z. Wu, Y. Jia, J. Chen, R. K. Zheng, Y. Zhang, H. Luo, Remanent-polarization-induced enhancement of photoluminescence in Pr^{3+}-doped lead-free ferroelectric $(Bi_{0.5}Na_{0.5})TiO_3$ ceramic. *Appl. Phys. Lett.* **102**, 042907 (2013).

13. H.-Y. Ye, Y.-Y. Tang, P.-F. Li, W.-Q. Liao, J.-X. Gao, X.-N. Hua, H. Cai, P.-P. Shi, Y.-M. You, R.-G. Xiong, Metal-free three-dimensional perovskite ferroelectrics. *Science* **361**, 151–155 (2018).

14. J. Rödel, W. Jo, K. T. P. Seifert, E.-M. Anton, T. Granzow, D. Damjanovic, Perspective on the development of lead-free piezoceramics. *J. Am. Ceram. Soc.* **92**, 1153–1177 (2009).

15. T. Handa, A. Wakamiya, Y. Kanemitsu, Photophysics of lead-free tin halide perovskite films and solar cells. *APL Mater.* **7**, 080903 (2019).

16. M. G. Ehrenreich, Z. Zeng, S. Burger, M. R. Warren, M. W. Gaultois, J. Tan, G. Kieslich, Mechanical properties of the ferroelectric metal-free perovskite $[MDABCO](NH_4)I_3$. *Chem. Commun.* **55**, 3911–3914 (2019).

17. D. J. W. Allen, N. C. Bristowe, A. L. Goodwin, H. H. M. Yeung, Mechanisms for collective inversion-symmetry breaking in dabconium perovskite ferroelectrics. *J. Mater. Chem. C* **9**, 2706–2711 (2021).

18. X. Song, Q. Cui, Y. Liu, Z. Xu, H. Cohen, C. Ma, Y. Fan, Y. Zhang, H. Ye, Z. Peng, R. Li, Y. Chen, J. Wang, H. Sun, Z. Yang, Z. Liu, Z. Yang, W. Huang, G. Hodes, S. (F.) Liu, K. Zhao, Metal-free halide perovskite single crystals with very long charge lifetimes for efficient x-ray imaging. *Adv. Mater.* **32**, 2003353 (2020).

19. K. Momma, F. Izumi, VESTA 3 for three-dimensional visualization of crystal, volumetric and morphology data. *J. Appl. Cryst.* **44**, 1272–1276 (2011).

20. T. Hahn, *International Tables for Crystallography: Volume A* (Springer, ed. 5, 2005).

21. R. W. Boyd, *Nonlinear Optics* (Academic Press, ed. 3, 2008).

22. S. A. Denev, T. T. A. Lummen, E. Barnes, A. Kumar, V. Gopalan, Probing ferroelectrics using optical second harmonic generation. *J. Am. Ceram. Soc.* **94**, 2699–2727 (2011).

23. T. W. Kasel, Z. Deng, A. M. Mroz, C. H. Hendon, K. T. Butler, P. Canepa, Metal-free perovskites for non linear optical materials. *Chem. Sci.* **10**, 8187–8194 (2019).

24. J. Bie, D.-B. Yang, M.-G. Ju, Q. Pan, Y.-M. You, W. Fa, X. C. Zeng, S. Chen, Molecular design of three-dimensional metal-free $A(NH_4)X_3$ perovskites for photovoltaic applications. *JACS Au* **1**, 475–483 (2021).

25. M. N. Kabler, Low-temperature recombination luminescence in alkali halide crystals. *Phys. Rev.* **136**, A1296–A1302 (1964).

26. M. N. Kabler, D. A. Patterson, Evidence for a triplet state of the self-trapped exciton in alkali-halide crystals. *Phys. Rev. Lett.* **19**, 652–655 (1967).

27. K. S. Song, R. T. Williams, *Self-Trapped Excitons* (Springer, ed. 2, 1996).

28. P. Y. Yu, M. Cardona, *Fundamentals of Semiconductors* (Springer, ed. 4, 2010).

29. H. Wang, H. Liu, Z. Zhang, Z. Liu, Z. Lv, T. Li, W. Ju, H. Li, X. Cai, H. Han, Large piezoelectric response in a family of metal-free perovskite ferroelectric compounds from first-principles calculations. *NPJ Comput. Mater.* **5**, 17 (2019).

30. M. Trassin, G. De Luca, S. Manz, M. Fiebig, Probing ferroelectric domain engineering in $BiFeO_3$ thin films by second harmonic generation. *Adv. Mater.* **27**, 4871–4876 (2015).

31. L. Wu, S. Patankar, T. Morimoto, N. L. Nair, E. Thewalt, A. Little, J. G. Analytis, J. E. Moore, J. Orenstein, Giant anisotropic nonlinear optical response in transition metal monopnictide Weyl semimetals. *Nat. Phys.* **13**, 350–355 (2017).

32. J. F. Nye, *Physical Properties of Crystals* (Oxford Univ. Press, 1957).

33. F. Zernike, J. E. Midwinter, *Applied Nonlinear Optics* (Wiley, 1973).

34. G. M. Sheldrick, *SHELX-97, Program for the Refinement of Crystal Structures* (University of Göttingen, 1997).

35. Y. Toyozawa, *Optical Processes in Solids* (Cambridge Univ. Press, 2003).
